# Bolus transit of upper esophageal sphincter on high-resolution impedance manometry study correlate with the laryngopharyngeal reflux symptoms

**DOI:** 10.1038/s41598-021-99927-0

**Published:** 2021-10-14

**Authors:** Jia-Feng Wu, Wei-Chung Hsu, I.-Jung Tsai, Tzu-Wei Tong, Yu-Cheng Lin, Chia-Hsiang Yang, Ping-Huei Tseng

**Affiliations:** 1 Departments of Pediatrics, National Taiwan University Hospital, Taipei, Taiwan, ROC; 2 Departments of Otolaryngology, National Taiwan University Hospital, Taipei, Taiwan, ROC; 3 Graduate Institute of Electronics Engineering, National Taiwan University, Taipei, Taiwan, ROC; 4 Department of Electrical Engineering, National Taiwan University, Taipei, Taiwan, ROC; 5 Department of Internal Medicine, National Taiwan University Hospital, Chung-Shan S. Rd, No. 7, Taipei, Taiwan, ROC

**Keywords:** Gastroenterology, Signs and symptoms

## Abstract

Laryngopharyngeal reflux symptom is a troublesome upper esophageal problem, and reflux symptom index (RSI) is commonly applied for the assessment of clinical severity. We investigated the relationship between the upper esophageal sphincter impedance integral (UESII) and RSI scores in this study. Totally 158 subjects with high-resolution esophageal impedance manometry (HRIM) with RSI questionnaire assessment were recruited. There are 57 (36.08%), 74 (46.84%), 21 (13.29%), and 6 (3.79%) patients were categorized as normal, ineffective esophageal motility disorder, absent contractility, and achalasia by HRIM examination, respectively. Subjects with RSI > 13 were noted to have lower UESII than others with RSI ≦ 13 (7363.14 ± 1085.58 vs. 11,833.75 ± 918.77 Ω s cm; P < 0.005). The ROC analysis yielded a UESII cutoff of < 2900 Ω s cm for the best prediction of subjects with RSI > 13 (P = 0.002). Both female gender and UESII cutoff of < 2900 Ω s cm were significant predictors of RSI > 13 in logistic regression analysis (OR = 3.84 and 2.83; P = 0.001 and 0.01; respectively). Lower UESII on HRIM study, indicating poor bolus transit of UES during saline swallows, is significantly associated with prominent laryngopharyngeal reflux symptoms scored by RSI score.

## Introduction

Esophageal dysmotility is a troublesome problem in both adults and children, and the current diagnosis is based on high-resolution esophageal manometry (HRM)^1,2^. Laryngopharyngeal symptom is considered one of the most difficult-to-diagnose manifestations of laryngopharyngeal and/or upper esophageal problems and may associate the function of the upper esophageal sphincter (UES)^3^. The clinical symptoms of laryngopharyngeal reflux disease (LPRD) include voice problem, throat clearing, the sensation of excess throat mucus, dysphagia, cough, breathing difficulties, troublesome or annoying cough, or lump sensation in the throat^4,5^.

Reflux symptom index (RSI), a nine-item self-administered outcome questionnaire, was developed to assess the symptoms and severity of laryngopharyngeal reflux symptoms and validated in follow-up studies^3–5^. A total RSI score of more than 13 is considered positive as far as diagnosis of LPRD^4,5^. However the diagnosis of LRPD remains difficult and the pathophysiology of the clinical symptoms of LPRD, especially the associated UES function, remains unknown in large^3–5^.

HRM and automatically calculated parameters, including the distal contractile integral (DCI), distal latency, and 4 s integrated relaxation pressure (IRP4s) to quantify the pressure changes in the esophagus, have been well developed to assist the differential diagnosis of various esophageal motility disorders^6,7^. The concomitant assessment of the esophageal intraluminal impedance signal is regarded as an effective modality for the assessment of bolus transit patterns in esophageal high-resolution impedance manometry (HRIM) study^8,9^. However, the current HRM study mainly assessed the function of the lower esophageal sphincter and esophageal body. The HRIM interpretation systems for assessing the bolus transit depend on the visual interpretation of impedance signals by clinical physicians^6–13^. A generalized automated analysis system to assess the UES motor function and bolus clearance remain needed. Assessing the UES pressure change and bolus transit by HRIM study may offer evidence of the pathophysiology of LPR symptoms.

In this study, we aimed to analyze the relationship between Novo parameters of UES (pressure and bolus clearance function) and the presence and severity of LPR symptoms in terms of RSI scores, and their possible roles for the automatic diagnosis of LPRD in the HRIM study.

## Materials and methods

### Study participants

We enrolled 158 subjects into this cohort for analysis between 2014 and 2020. There are 101 consecutive patients with various esophageal symptoms such as reflux, dysphagia, and globus sensation (53.40 ± 15.85 years; 38 males and 63 females) and another 57 adult asymptomatic health control subjects (age 41.54 ± 11.75 years; 32 males and 25 females) receiving the HRIM study and RSI questionnaire at the same day in the gastrointestinal motility laboratory of National Taiwan University Hospital into this analysis. The interpretation of the manometric parameters of HRM was based on the Chicago Classification version 3.0 criteria^6^. The anthropometric data including the body height and weight of the study subjects were also collected. The study protocol was approved by the Institutional Review Board of National Taiwan University Hospital. The written informed consent was obtained from all patients for the HRIM examination and RSI questionnaire assessment in this study. The study was conducted following the principles of the Declaration of Helsinki and the International Conference on Harmonization for Good Clinical Practice.

### HRIM study and data interpretation

In this study cohort, all subjects were instructed to maintain a nil per os status for at least 8 h before the HRIM examination. The HRIM examination in this study was performed using a 4.2-mm-diameter silicone catheter with 22 closely spaced water-perfused pressure sensors and 12 impedance channels (PART#CE4-1083; Dentsleeve International Ltd., Ontario, Canada) as described in our previous study^13^. The side holes of the HRIM catheter were perfused with distilled water at a rate of 0.15 mL per min using a pneumatic perfusion pump throughout the manometric study, and the pressure/impedance data was recorded using external pressure transducers (Solar GI HRIM water-perfused system, Medical Measurement Systems, Enschede, Netherlands).

All subjects were instructed to take 10 liquid swallows of 5 mL saline at 30 secs intervals after the successful HRIM catheter insertion. The HRIM pressure and impedance signals were recorded at a frequency of 20 Hz and stored on a personal computer. The MMS HRIM software converts recorded signals into digital data, which are displayed as color plots on the Solar GI HRM Compact Pole system (version 9.5, MMS, Solar GI HRIM water-perfused system, Medical Measurement Systems, Enschede, Netherlands).

### Quantification of the upper esophageal sphincter impedance integral (UESII)

**Figure 1:**
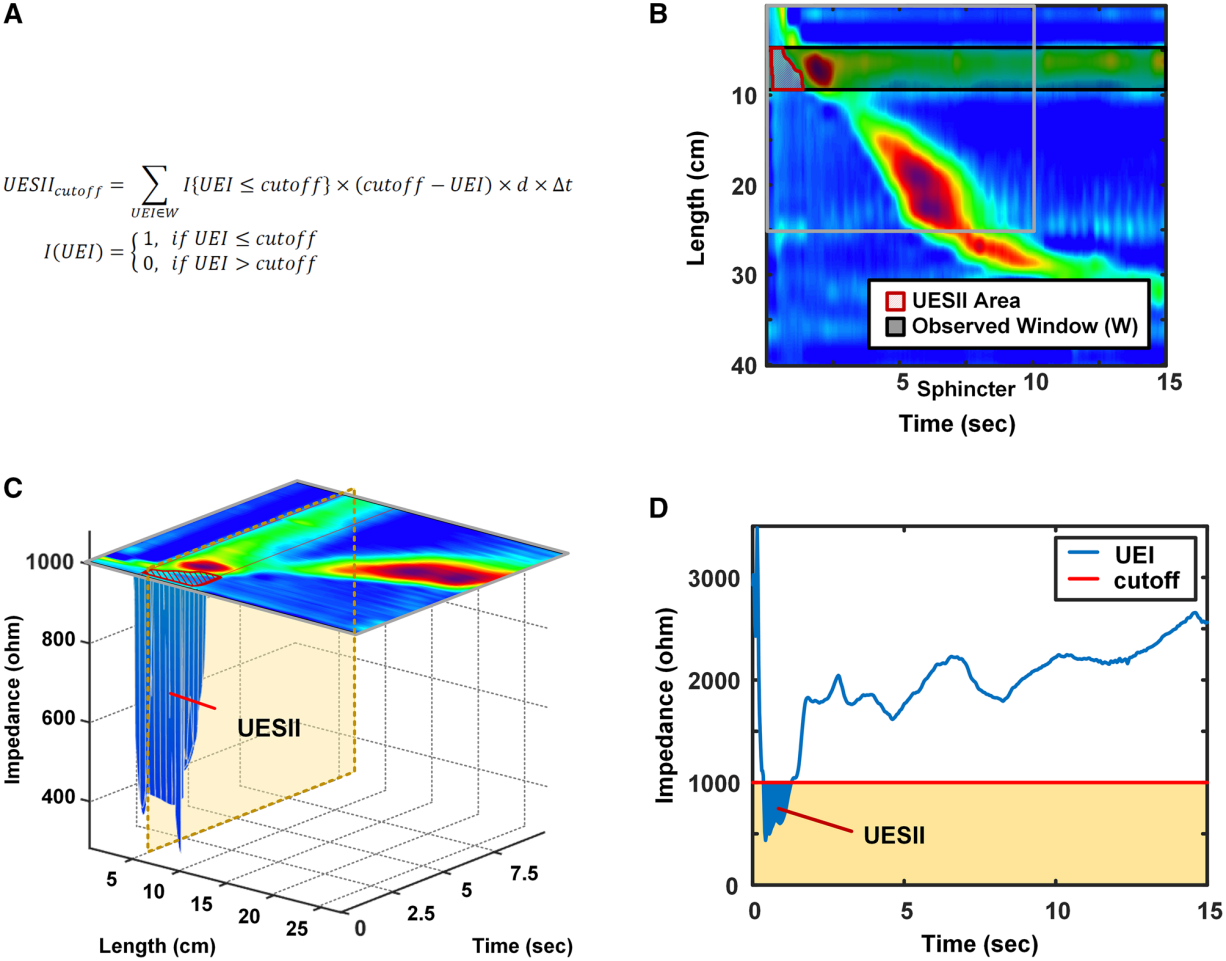
(**A**) In the formula of upper esophageal sphincter impedance integral (UESII), the “*UEI*” means upper esophageal impedance, “*d*” means the distance between two adjacent impedance channels, “*Δt*” is the sampling interval, “*W*” is the observed window, and “*I*” is the indicator function. (**B**) The 2D plot of UESII of the wet swallow. (**C**) The 3D plot of UESII of the wet swallow was demonstrated. (**D**) Calculation of the UESII value at less than 1000 Ω.

We calculated the UESII below impedance thresholds of 1000Ω over an observed time window of 15 s to quantify the bolus transit signal of UES during the 5-mL saline liquid swallow test (Fig. 1A–D). In the formula (Fig. 1A), the “*UEI*” means upper esophageal impedance, “*d*” is the distance between two adjacent impedance channels for “*UEI*”, “*Δt*” is the sampling interval of the sensors, “*W*” is the observed window, and “*I*” is the indicator function. The mean UESII of 10 liquid swallows in each subject was evaluated in the statistical analyses.

### Quantification of the upper esophageal sphincter relaxation integral (UESRI)

**Figure 2:**
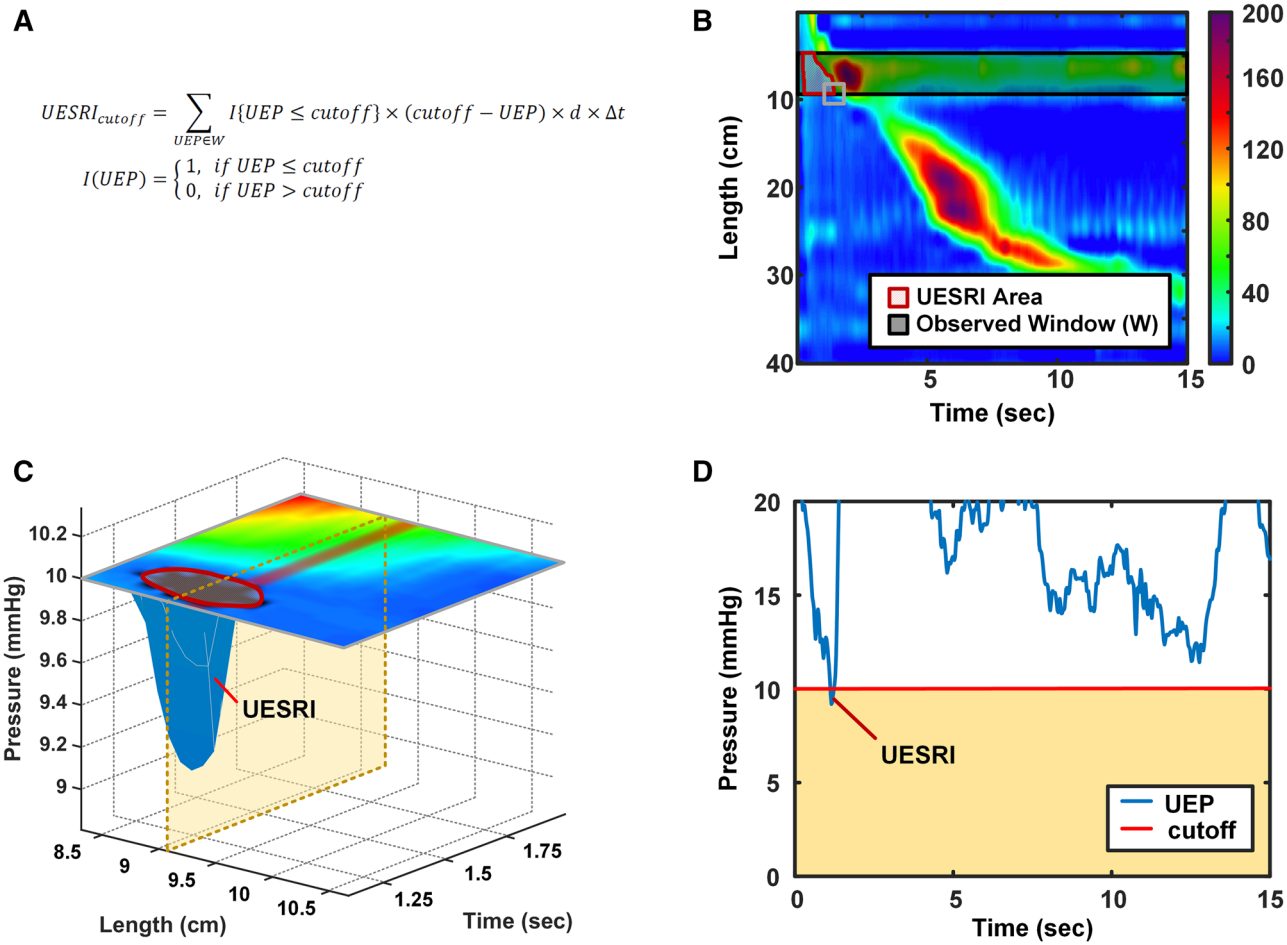
(**A**) In the formula of upper esophageal sphincter relaxation integral (UESRI), the “*UEP*” means upper esophageal impedance, “*d*” means the distance between two adjacent pressure sensors, “*Δt*” is the sampling interval, “*W*” is the observed window, and “*I*” is the indicator function. (**B**) The 2 D plot of UESRI of the wet swallow. (**C**) The 3D plot of UESRI of the wet swallow. (**D**) Calculation of the UESRI value at less than 10 mmHg.

The UESRI below pressure thresholds of 10 mmHg over an observed time window of 15 s to quantify the motor function of UES relaxation during the 5-mL saline liquid swallow test was calculated (Fig. 2A–D). In the formula (Fig. 2A), “*UEP*” means upper esophageal pressure, “*d*” is the distance between two adjacent impedance channels for “*UEI*” or two adjacent pressure sensors for “*UEP*”, “*Δt*” is the sampling interval of the sensors, “*W*” is the observed window, and “*I*” is the indicator function. The mean UESRI of 10 liquid swallows in each subject during HRIM examination was evaluated in the statistical analyses.

### Symptom questionnaires evaluation

All study subjects were assessed for gastrointestinal symptoms by using validated RSI symptom questionnaires on the same day of HRIM study^3^. The RSI score > 13 is used for the clinical diagnosis of LPRD^3^. The total RSI score, and the 9 individual RSI sub-scores are included in the data analysis.

### Statistical analysis

The MedCalc (version 20.09; MedCalc Software, Ostend, Belgium) and STATA (version 14.2; StataCorp LP, College Station, TX, USA) software were applied for the statistical analyses in this study. The MATLAB software (version 8.6 R2015b; MathWorks, Natick, MA, USA) was used to quantify the UESRI, and UESII in this study. Fisher’s exact test or the chi-squared test was used to determine differences in categorical variables between the groups. Student’s t-test with unequal variance was applied to assess differences in the mean, standard error (SE), and 95% confidence interval (CI) values of the continuous variables between groups. Correlation analysis between RSI score and UES metrics was analyzed. Univariate and multivariate logistic regression analyses were also performed. Receiver operating characteristic (ROC) analysis was performed to determine cutoff values for predicting RSI > 13 and their respective area under the curve (AUC) values. The sensitivity, specificity, positive predictive value (PPV), negative predictive value (NPV), and diagnostic accuracy of each diagnostic parameter were also analyzed. A P value < 0.05 was regarded as indicative of statistical significance.

## Results

### General characteristics

Among these 101 consecutive patients with various esophageal symptoms, 74 (73.27%), 21 (20.79%), and 6 (5.94%) patients were categorized as ineffective esophageal motility disorder, absent contractility, and achalasia (2 type I, 3 type II, and 1 type III achalasia patients) according to the Chicago Classification version 3.0 criteria, respectively. All healthy control (n = 57) had normal esophageal manometry data. There are 48 (30.38%) subjects with self-reporting RSI score > 13 on the same day of HRIM study in this study cohort (Table 1).

**Table 1:** General characteristics of the study cohort.

	Study cohort (n = 158)
Male gender, n (%)	70 (44.30%)
Age, mean ± SE (years)	49.13 ± 1.24
Body weight, mean ± SE (Kg)	61.28 ± 1.20
Height, mean ± SE (m)	1.63 ± 0.01
Body mass index, mean ± SE (kg/m2)	22.94 ± 0.33
Waist, mean ± SE (cm)	80.30 ± 0.94
**High resolution esophageal manometry finding**	
Normal, n (%)	57 (36.08%)
Ineffective esophageal motility disorder, n (%)	74 (46.84%)
Absent contractility, n (%)	21 (13.29%)
Achalasia, n (%)	6 (3.79%)
**Reflux symptom index (RSI)**	
RSI score > 13, n (%)	48 (30.38%)
RSI score ≦ 13, n (%)	110 (69.62%)
Upper esophageal sphincter relaxation integral (UESRI), mmHg s cm	2175.69 ± 311.24
Upper esophageal sphincter impedance integral (UESII), Ω s cm	10,475.59 ± 736.28

The RSI score is significantly higher in subjects with esophageal motility disorders (ineffective esophageal motility disorders, absent contractility, and achalasia) than others with normal esophageal manometry data (13.90 ± 1.08 vs. 0.53 ± 0.17; 95% CI 11.76–16.05 vs. 0.19–0.85; P < 0.0001). The percentage of female subjects in the RSI score > 13 group (n = 48) is significantly higher than that in the RSI score ≦ 13 group (n = 110) in this cohort (79.16% vs. 46.45%; P < 0.001). Subjects with RSI score > 13 (n = 48) were also noted to have lower UESII than others with RSI score ≦ 13 (n = 110) (7363.14 ± 1085.58 vs. 11,833.75 ± 918.77 Ω s cm; 95% CI, 5179.24–9547.05 vs. 10,012.78–13,654.71 Ω s cm; P < 0.005). In this cohort, RSI > 13 was found in 47.30% (35/74) IEM patients, 16.67% (1/6) achalasia patients, and 57.14% (12/21) patients with absent contractility, and 0% (0/57) subjects with normal esophageal manometry (P < 0.001).

There is no significant difference in UESRI between subjects with RSI score > 13 and ≦13 (1680.35 ± 274.75 vs. 2391.81 ± 429.88 mmHg s cm; 95% CI, 1127.62–2233.07 vs. 1539.796- 3243.82 mmHg s cm; P = 0.29).

### The UES metrics in subjects with normal HRIM

The mean UESII, indicating the bolus transit of UES, in subjects with normal esophageal manometry is 14,095.36 Ω s cm (95% CI, 11,889.32–16,301.41 Ω s cm). The mean UESRI, indicating the pressure change of UES during 5 mL liquid swallow, is 1490.95 mmHg s cm (95% CI, 1128.69–1853.21 mmHg s cm) in subjects with normal esophageal manometry.

### Relationship between RSI and UES metrics

The UES bolus transit metric, UESII, was demonstrated to negatively correlate with the total RSI score in this study cohort (P = 0.005, Table 2). The UESII was further demonstrated to correlate negatively with 6 of the 9 RSI sub-score items (including hoarseness or a problem with your voice, clearing your throat, difficulty swallowing food/liquids/pills, breathing difficulties or chocking episode, the sensation of something sticking in your throat or a lump in your throat, and heartburn/chest pain/indigestion/stomach acid coming up) in the study cohort (P < 0.05, Table 2). There is no significant statistical difference between UESRI and RSI total/sub-score items in this study (Table 2).

**Table 2:** Correlation between upper esophageal sphincter impedance integral (UESII), and upper esophageal sphincter relaxation integral (UESRI) with reflux symptom index (RSI).

	UESII		UESRI	
	Correlation coefficient	P value	Correlation coefficient	P value
Total RSI score	− 0.22	0.005	− 0.09	0.27
**RSI sub-score**				
Hoarseness or a problem with your voice	− 0.26	0.001	− 0.01	0.93
Clearing your throat	− 0.16	0.04	− 0.09	0.28
Excess throat mucus or postnasal drip	− 0.10	0.23	− 0.10	0.20
Difficulty swallowing food, liquids, pills	− 0.23	0.004	0.01	0.86
Coughing after you ate or after lying down	− 0.10	0.19	− 0.09	0.26
Breathing difficulties or chocking episode	− 0.19	0.02	− 0.01	0.87
Troublesome or annoying cough	− 0.10	0.23	− 0.09	0.25
Sensation of something sticking in your throat or a lump in your throat	− 0.21	0.007	− 0.10	0.19
Heartburn, chest pain, indigestion, or stomach acid coming up	− 0.18	0.02	− 0.06	0.46

### Predictors of RSI score > 13 in this study population

**Figure 3:**
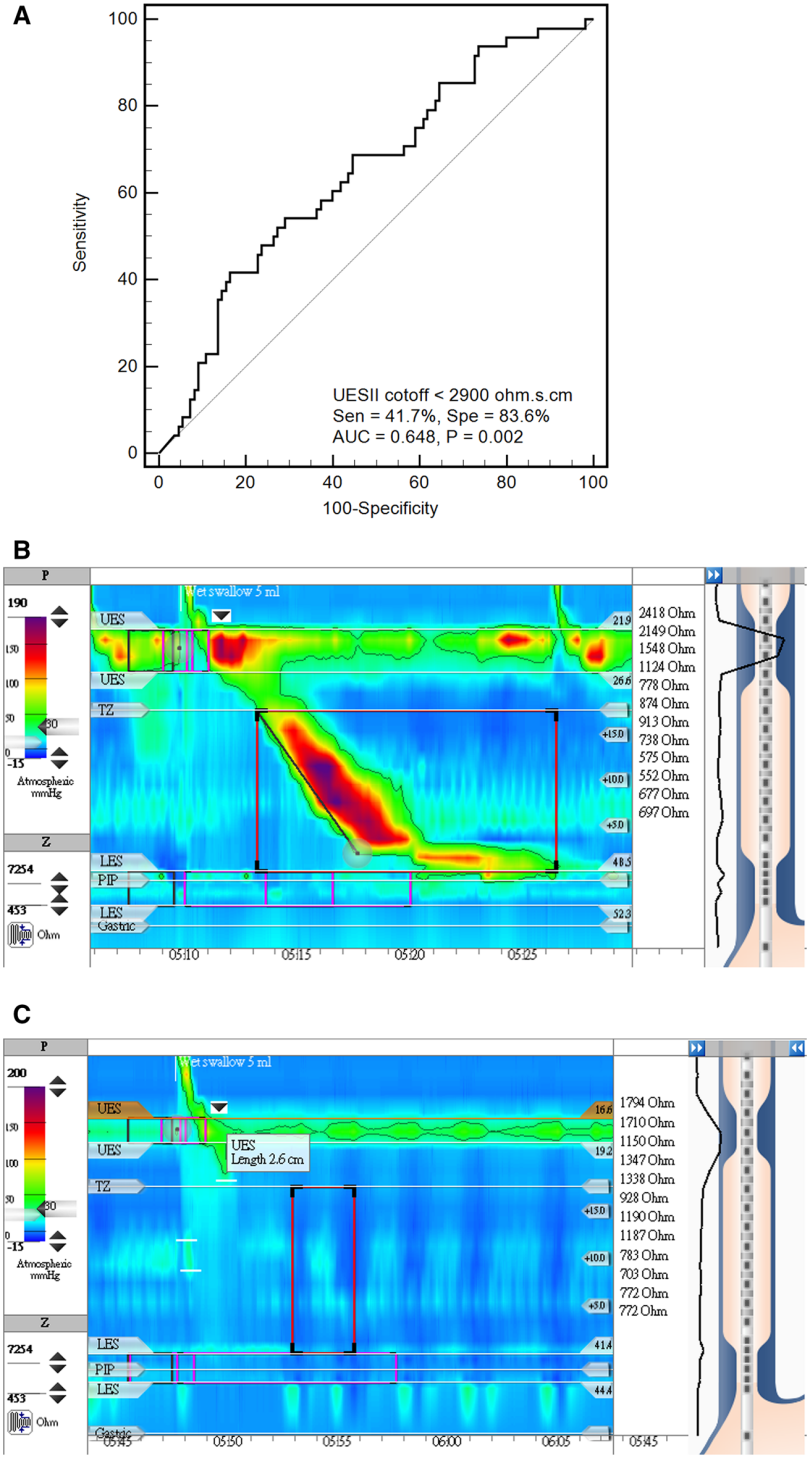
(**A**) The ROC analysis indicated that a upper esophageal sphincter impedance integral (UESII) cutoff of 2900 Ω s cm achieved the best differentiation between subjects with reflux symptom index (RSI) score > 13 and others with RSI score ≦ 13. (**B**) A subject with RSI score = 0 had the impedance value of 2418 Ω above upper esophageal sphincter immediate after 5 mL saline liquid swallow. (**C**) A subject with RSI score = 42 had the impedance value of 1794 Ω above upper esophageal sphincter immediate after 5 mL saline liquid swallow.

The ROC analysis revealed that a UESII cutoff of 2900 Ω s cm had the best ability to differentiate subjects with RSI score > 13 from others with RSI score ≦ 13 (AUC 64.8%, P = 0.002, Fig. 3A). The PPV, NPV and diagnostic accuracy for this cutoff (UESII < 2900 Ω s cm) to predict RSI score > 13 are 52.63%, 76.67%, and 70.89%, respectively.

There were 35.14% (26/74) of ineffective esophageal motility disorder patients, 66.67% (4/6) of achalasia patients, 28.57% (6/21) of absent contractility patients, and 3.51% (2/57) of normal manometry subjects with UESII < 2900 Ω s cm (P < 0.001).

In the univariate logistic regression analysis models, both female gender and UESII < 2900 Ω s cm were significant predictors for the prediction of RSI score > 13 in this study cohort (OR = 4.56 and 3.65; P < 0.001 and = 0.001, respectively, Table 3).

**Table 3:** Predictors of reflux symptom index (RSI) > 13 in this study cohort analyzed by univariate and multivariate logistic regression model.

	Univariate analysis			Multivariate analysis		
	OR	95% CI	P	OR	95% CI	P
Female vs. Male (n = 88 vs. 70)	4.56	2.07–10.06	< 0.001	3.84	1.71–8.63	0.001
UESII < 2900 vs. ≧ 2900 Ω s cm (n = 38 vs. 120)	3.65	1.70–7.84	0.001	2.83	1.27–6.30	0.01

The UESII < 2900 Ω s cm remained a significant predictor of RSI score > 13 after the adjustment of gender in the multivariate logistic regression model (OR = 2.83; 95% CI = 1.27–6.30; P = 0.01; Table 3).

Subjects with a high RSI score also had a lower impedance value above UES immediately after liquid swallow than others with a low RSI score (Fig. 3B,C). The evidence suggested that subjects with higher RSI scores may have the problem of UES bolus transit during liquid swallows, causing fluid retention above UES after the closure of UES.

## Discussion

The exact pathophysiology of LPRD remains unclear in large to date, and the proposed mechanism includes high gastroesophageal reflux, the impairment of the subjective and objective voice quality evaluations, and even psychological problems^14–20^. Belafsky et al. developed a nine-item questionnaire (RSI) for the assessment of symptoms in LPRD patients, and subjects with a total RSI score of more than 13 is considered diagnostic of LPRD^3–5^. There remains no gold standard for the diagnosis of LPRD to date^6,7^.

However, only a few LPRD patients were confirmed to have high gastroesophageal reflux assessed by multichannel intraluminal esophageal impedance-pH monitoring previously^5^. Recent studies proposed the application of multichannel intraluminal esophageal impedance-pH monitoring, pepsin, and bile salt detection to assist the diagnosis of LPRD, but the diagnostic performance of these tools remains unclear^5,18^. All of these diagnostic modalities focus on high gastroesophageal reflux-related laryngopharyngeal reflux symptoms^5,18^. Other than RSI clinical symptom score, there is no validated diagnostic test to confirm LPRD to date^5,18,19^.

Current treatment options for LPRD include dietary measures, proton pump inhibitors, alginate, and magaldrate^18^. But the efficacy of anti-reflux and acid-suppression agents to relieve laryngopharyngeal reflux symptoms remains controversial^18,21–23^. The reported success rate of conventional therapy ranged from 17 to 87%, and the treatment outcomes varied substantially between studies^20,21^. Psychological problems are usually considered in LPRD patients with sub-optimal clinical responses to conventional therapeutic agents^14,16^. The low treatment success rate of conventional therapy is highly possible to associate with the diversity of LPRD, and the non-reflux mechanism of laryngopharyngeal reflux symptoms may play roles in part of these patients.

A recent study demonstrated abnormal findings on HRM identified in 43.3% of patients with LPR symptoms, but there remain 56.7% of subjects with LPR symptoms without obvious abnormal findings based on current HRIM interpretation criteria^23^. The data implying the important role of routine HRM study in patients with LPR symptoms to evaluate the possibility of an esophageal motility disorder^23^. However, there are no HRIM criteria established to assist the confirmation of laryngopharyngeal symptoms in subjects with normal manometry data graded by current guidelines^6,7,13^.

Since the majority of laryngopharyngeal symptoms scored by RSI questionnaire are located above UES^3^. We firstly demonstrated that the UES motility function may associate with the laryngopharyngeal reflux symptoms, and thus quantify the UES metrics (UES pressure change by UESRI, and UES bolus transit by UESII) in this study. Poor bolus transit of UES, indicating by lower UESII, is associated with a higher RSI total score and the majority of RSI sub-scores (66.7%) in our study.

Our data showed further subjects with a high RSI score had a lower impedance value above UES immediately after liquid swallow than others with a low RSI score. The evidence indicating subjects with high RSI scores have the problem of fluid retention above UES after the contraction of UES. These non-swallow foods or liquid above UES after swallow can induce laryngopharyngeal reflux symptoms such as hoarseness, clearing throat, difficulty in swallowing food/liquids/pills, choking episode, and lump sensation of the throat. Hence, gastroesophageal reflux maybe not be the sole etiology for laryngopharyngeal reflux symptoms. UES bolus transit failure may be responsible for part of the mechanism of laryngopharyngeal reflux symptoms. The therapeutic management to improve UES bolus transit may help to relieve laryngopharyngeal reflux symptoms, especially for those with inadequate response to conventional anti-reflux therapeutic agents.

There are possible limitations of our study. Our cohort is recruited between 2014 and 2020, and all HRIM data were analyzed according to version 3.0 of Chicago Classification at the time of HRIM study. We re-assessed our HRIM data according to version 4.0 of Chicago Classification, which is published in 2021, and only the number of the diagnosis of IEM has changed (from 74 in version 3.0 to 44 in version 4.0 of Chicago Classification) while the diagnosis of other esophageal motility disorders remains unchanged. There is no statistic difference between IEM subjects fulfill both version 3.0 and 4.0 Chicago Classification (n = 44) and IEM subjects only fulfill version 3.0 Chicago Classification (n = 30) in total RSI score (13.48 ± 1.47 vs. 15.03 ± 2.16, P = 0.54) and UESII value (8118.16 ± 1354.73 vs. 9442.67 ± 1784.51 Ω s cm, P = 0.58). Since our study only assessed the pressure and bolus change of UES, version of the Chicago Classification will not alter the results and conclusions of the current study. Our study demonstrated the bolus transit of UES (UESII) is associated with RSI score, but not the pressure changes of UES (UESRI) during 5 mL saline liquid swallow of the HRIM study. Further larger-scale studies, with larger statistical power, are still needed in the future to confirm this phenomenon. Different HRIM catheters may result in different measurements, and further studies applying other design HRIM catheters remain needed. Water-perfused manometry system used in the present study has been validated for the assessment of UES and esophageal bolus transit function in previous studies^13,24–26^. The side holes of the HRIM catheter were perfused with distilled water at a rate of 0.15 mL per min throughout the manometric study in our institute. Since distilled water perfused from the catheter is electrolyte-free, we believe it had a minimal impact of the impedance measurement on our subjects.

In conclusion, our findings suggest that the novel parameter UESII, which indicates the bolus transit of UES of liquid swallow, is correlated well with RSI symptom scores. This novel parameter may have a role in explaining the physiology and its clinical consequences in clinical symptoms of laryngopharyngeal reflux. It may also serve as an adjunct parameter of UES in HRIM study, and possibly help the clinicians to make stratified therapeutic decisions in the future.

## Data Availability

Data maybe available after the approval of IRB of NTUH.

## Abbreviations

HRIM: High-resolution esophageal impedance manometry
LPRD: Laryngopharyngeal reflux disease
RSI: Reflux symptom index
UESII: Upper esophageal sphincter impedance integral
UESRI: Upper esophageal sphincter relaxation integral
